# Quantifying phenological diversity: a framework based on Hill numbers theory

**DOI:** 10.7717/peerj.13412

**Published:** 2022-05-12

**Authors:** Daniel Sánchez-Ochoa, Edgar J. González, Maria del Coro Arizmendi, Patricia Koleff, Raúl Martell-Dubois, Jorge A. Meave, Hibraim Adán Pérez-Mendoza

**Affiliations:** 1 Laboratorio de Ecología Evolutiva y Conservación de Anfibios y Reptiles, Facultad de Estudios Superiores Iztacala, Universidad Nacional Autónoma de México, Tlalnepantla de Baz, México, Mexico; 2 Posgrado en Ciencias Biológicas, Unidad de Posgrado, Circuito de Posgrados, Universidad Nacional Autónoma de México, Ciudad Universitaria, Coyoacán, Ciudad de México, Mexico; 3 Departamento de Ecología y Recursos Naturales, Facultad de Ciencias, Universidad Nacional Autónoma de México, Ciudad Universitaria, Coyoacán, Ciudad de México, Mexico; 4 Laboratorio de Ecología, UBIPRO, Facultad de Estudios Superiores Iztacala, Universidad Nacional Autónoma de México, Tlalnepantla de Baz, México, Mexico; 5 Comisión Nacional para el Conocimiento y Uso de la Biodiversidad, Tlalpan, Ciudad de México, Mexico

**Keywords:** Biodiversity, Dissimilarity, Hill numbers, Overlap index, Phenology, Time series analysis

## Abstract

**Background:**

Despite the great concern triggered by the environmental crisis worldwide, the loss of temporal key functions and processes involved in biodiversity maintenance has received little attention. Species are restricted in their life cycles by environmental variables because of their physiological and behavioral properties; thus, the timing and duration of species’ presence and their activities vary greatly between species within a community. Despite the ecological relevance of such variation, there is currently no measure that summarizes the key temporal aspects of biological diversity and allows comparisons of community phenological patterns. Here, we propose a measure that synthesizes variability of phenological patterns using the Hill numbers-based attribute diversity framework.

**Methods:**

We constructed a new phenological diversity measure based on the aforementioned framework through pairwise overlapping distances, which was supplemented with wavelet analysis. The Hill numbers approach was chosen as an adequate way to define a set of diversity values of different order q, a parameter that determines the sensitivity of the diversity measure to abundance. Wavelet transform analysis was used to model continuous variables from incomplete data sets for different phenophases. The new measure, which we call Phenological Hill numbers (PD), considers the decouplings of phenophases through an overlapping area value between pairs of species within the community. PD was first tested through simulations with varying overlap in phenophase magnitude and intensity and varying number of species, and then by using one real data set.

**Results:**

PD maintains the diversity patterns of order q as in any other diversity measure encompassed by the Hill numbers framework. Minimum PD values in the simulated data sets reflect a lack of differentiation in the phenological curves of the community over time; by contrast, the maximum PD values reflected the most diverse simulations in which phenological curves were equally distributed over time. PD values were consistent with the homogeneous distribution of the intensity and concurrence of phenophases over time, both in the simulated and the real data set.

**Discussion:**

PD provides an efficient, readily interpretable and comparable measure that summarizes the variety of phenological patterns observed in ecological communities. PD retains the diversity patterns of order q characteristic of all diversity measures encompassed by the distance-based Hill numbers framework. In addition, wavelet transform analysis proved useful for constructing a continuous phenological curve. This methodological approach to quantify phenological diversity produces simple and intuitive values for the examination of phenological diversity and can be widely applied to any taxon or community’s phenological traits.

## Introduction

The biodiversity crisis involves the loss of species and their functions from all ecosystems worldwide (Chen & Shen, 2017). The loss of key temporal functions and processes involved in biodiversity maintenance is a major component of biodiversity loss (Lamy et al., 2015; Cardinale et al., 2012; Youngflesh et al., 2021). Despite the great concern triggered by this crisis, a measure for summarizing the diversity of temporal patterns in communities has received little research (Legendre & Gauthier, 2014; Legendre, 2019). Phenology is defined as the study of the timing of recurring biological events, their variation within and among species, and the biotic and abiotic agents responsible for the initiation, duration, and end of such events (Paranjpe & Sharma, 2005; Liang & Schwartz, 2009; Lieth, 1974; Denny et al., 2014). The term phenology also refers to the timing of recurrent biological events themselves (*i.e.,* phenological patterns) (Hudson, 2010; Wolkovich, Cook & Davies, 2014; Cohen, Lajeunesse & Rohr, 2018). Under this latter conceptualization, phenology has been interpreted as an expression of the environmental responses of organisms over time. Species are generally restricted in their life cycle by environmental variables because of their physiological and behavioral traits. Consequently, the timing and duration of phenophases (*e.g.*, the distinguishable portion or aspect of an organism’s life cycle) vary greatly among species within a community (Angus & Moncur, 1977; Bunting, 1975; Visser & Both, 2005; Cleland et al., 2007; Stange & Ayres, 2010; Gompper et al., 2016; Horne, 2017; Tonkin et al., 2017; Eisenhauer et al., 2018; Li et al., 2018). Despite the high degree of variation in phenophases among species in a community and the ecological significance of this variation, a measure summarizing the diversity in the timing of biological events in communities is still lacking.

Given the differences in the types of phenological events, variation within species, and life histories, approaches for measuring phenology are many-fold, which makes generalizing phenology complex (*i.e.,* depending on their life cycle and whether they are unitary or modular; Vuorisalo & Tuomi, 1986). Phenological data come in numerous forms, and the phenophases of organisms are often measured using presence/absence data and intensity or abundance counts of distinguishable biological processes or activities displayed by individuals in a particular timeframe (*e.g.*, growing, feeding, courtship, mating, breeding). Non-sessile organisms may respond rapidly to environmental variation by altering their behavior and activity patterns over time; in addition, capture and handling may be required to distinguish the phenophases of these organisms (*e.g.*, Tang et al, 2016; Marra et al., 2005; García-Cobos, Crawford & Ramírez-Pinilla, 2020). By contrast, in most modular and sessile organisms (mostly plants but also sessile animals, particularly benthic ones), phenophases are easily distinguishable among individuals along their life cycle, such as flowering, leaf abscission, and budding (Denny et al., 2014).

Phenological patterns can be studied at different biological levels of organization, including individuals, populations, and communities (Denny et al., 2014). The study of phenology at the individual level usually involves analyses of the relationship between phenological events and environmental cues (*e.g.*, Marchand et al., 2020). However, environmental relationships have also been examined at the population level, and synchronicity indices have been developed to analyze the variation in the timing of phenophases among individuals within a single population or between populations (*e.g.*, Hodges & Doraiswamy, 1979; Kharouba et al., 2018; Ghosh et al., 2020; Michel et al., 2020; Félix-Burruel et al., 2021). At the community level, synchronicity measures have been used to explore the degree of overlap in the timing of phenophases among few species or functional groups (*e.g.*, Heithaus, 1979; Herrero, 2003; Bartomeus et al., 2013; Corredor, 2020; McDevitt-Galles et al., 2020; Ramakers, Gienapp & Visser, 2020). Some community-level phenological studies have used descriptive approaches to analyze species phenophases and explore the environmental drivers of phenological patterns (Slagsvold, 1977; Ovaskainen et al., 2013). Phenology has been widely studied, but there is currently no measure that permits comparisons between communities to be made. Therefore, a measure is necessary for summarizing the temporal axis of biological diversity is needed to facilitate comparisons of phenologies between communities and generate new ecological questions.

Within-community phenological variation can be summarized based on the magnitude of the decouplings between the phenophases displayed by different species within a community, including their timing, duration and intensity. Thus, a measure of the differences among the phenological patterns might be indirectly correlated with the biotic interactions that shape phenological patterns (Walker & Chapin, 1987; Thorn et al., 2020; Visser & Both, 2005; Cleland et al., 2007; Stange & Ayres, 2010; Inouye, Ehrlén & Underwood, 2019; Gompper et al., 2016; Horne, 2017; Tonkin et al., 2017; Eisenhauer et al., 2018; Li et al., 2018), improving our understanding of how species or communities respond to both biotic and abiotic environmental cues (Losey & Denno, 1999; McKinney & Goodell, 2011; Bertness et al., 2014; Chuine & Régnière, 2017). Such a measure that incorporates phenological shifts between species and indirectly involves positive and negative interspecific interactions due to temporal niche overlap can provide insight into processes of facilitation or limitation based on the afforementioned decouplings in the phenophases of co-occurring species (Bergamo et al., 2018; Hegland et al., 2009; Jönsson et al., 2009; Forrest & Miller-Rushing, 2010). Given that diversity measures summarize key ecological aspects of communities, they are the basis for improving our understanding of more complex and specific ecological phenomena such as the large-scale consequences of climate change on communities (Pau et al., 2011; Pérez-Ramos et al., 2020; Alp et al., 2016).

Here, we develop a novel measure that summarizes a key aspect of temporal biological diversity: phenological Hill numbers (PD). We define PD as the variety of phenological patterns observed in ecological communities over a defined time period. Accordingly, PD reflects the distribution of temporal niches of the species occurring in the community and capture its relationship with the environment (including the indirect responses to biological interactions).

## Material and Methods

We constructed our phenological diversity measure using the Hill numbers-based attribute diversity framework (Chiu & Chao, 2014; Chao, Chiu & Jost, 2014) for phenological intensity/abundance data. Although this framework is based on pairwise overlapping distance, long-term phenological sampling often produces information gaps in the estimates of discrete variables and results in abrupt changes over time. This inconvenience can be solved through wavelet transform analysis, which is a reliable approach for predicting values to fill these gaps and model a continuous variable (Jung & Tremayne, 2003; Tuljapurkar & Haridas, 2006; Wei, 2006; Bradshaw & Spies, 1992; Frick, Grossmann & Tchamitchian, 1998; Mondal & Percival, 2010).

### Phenology as a continuous variable: wavelet time series analysis

**Figure 1:**
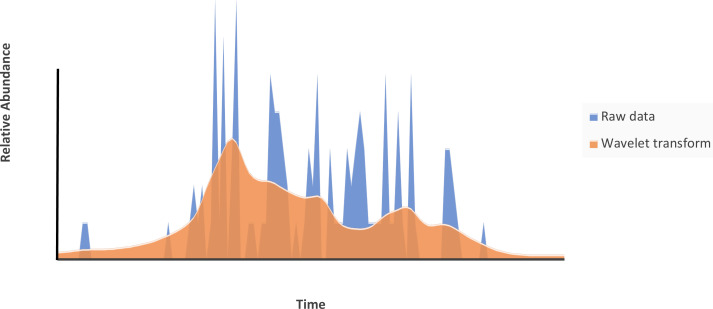
Comparison of two phenological curves for the same data set. The blue curve represents the raw data, and the red curve represents the smoothed phenological curve through the wavelet transform.

Wavelet transform detects the frequency spectrum of discrete time series data and fits a smoothed curve to them (Schmidt & Skidmore, 2004; Sifuzzaman, Islam & Ali, 2009). This analysis is based on the comparison of the similarity between a scaling function (which can be stretched, shrunk, and shifted in time) and the original time series (Torrence & Compo, 1998). With these comparisons, a matrix is constructed that contains the fits of the scaling function to the time series, in which the total sum of each column in the matrix produces the smoothed curve. In the case of phenological data, we can use such a smooth curve as an approximation to a continuous phenological pattern, in which the abrupt changes caused by sampling effort and protocol are smoothed out, in line with the assumption that phenophases start and finish gradually rather than abruptly (Fig. 1).

Wavelet transform analyses require specifying two parameters. The first parameter is the scaling function. The most common scaling functions are Morlet, Paul, DOG, Biorthogonal and Mexican hat*,* and these differ mainly from each other in the signal resolution calculation (González-Nuevo et al., 2006; Singh, Singh & Sharma, 2011). For our purposes, the Morlet scaling function is preferable, as it is optimal for data that cannot be directly interpreted, and time series with unknown frequencies and scales (Percival & Walden, 2000). The second parameter is the attenuation threshold (*τ*), which determines the phenological curve steepness. Values close to 0 translate into a highly smoothed curve; conversely, values approaching infinity translate into a wiggly curve, similar to the raw time series data. A value of 2 is used as a standard for wavelet analysis in mathematics software (*e.g.*, Matlab and wavScalogram R package). Wavelet analysis must be performed on the discrete phenological curve or time series of each studied species in the community; thus, the number of total smoothed phenological curves that must be calculated equals the number of species in the community (*S*)*.*

### Quantifying Phenological Hill numbers (PD)

Our phenological diversity measure is based on the Hill numbers-based attribute diversity framework (Jost, 2007; Chiu & Chao, 2014; Chao, Chiu & Jost, 2014). Hill numbers are a set of metrics that have two major advantages over other diversity indices: (1) the interpretation of the diversity values are consistently the same, and (2) the sensitivity regarding abundant and rare species or traits can be regulated with a parameter (*q*). This *q* parameter directly determines the sensitivity of the diversity measure (^*q*^*D*) to the relative abundances of species occurring in the community. Although *q* can take any non-negative real number, ecologists typically consider three values: 0, 1 and 2 (Chao, Chiu & Jost, 2014). When *q* = 0, the relative abundance of species is overlooked, and the measure simply represents *S* (*i.e.,* ^0^*D* =* S*). When *q* = 1, *S* is weighted by the proportions of the species abundances and can be interpreted as the effective number of species equally abundant within an assemblage, which is equivalent to exp(*H) (*i.e.*,* the exponent of Shannon’s entropy; Jost, 2006). Finally, when *q* = 2, the diversity values favor the most abundant species, and the less abundant or rare species are almost not accounted for; consequently, ^2^*D* can be roughly interpreted as the effective number of dominant or the most abundant species in the community (Jost, 2006). In addition, Hill numbers are consistent with basic diversity concepts like evenness and dominance (Chiu & Chao, 2014; Chao, Chiu & Jost, 2014). Additionally, Hill numbers are expressed in intuitive units of effective number of species, and they can be directly compared across orders of *q* to gain information on the dominance, community traits and comparisons among different species assemblages. Finally, Hill numbers theory can be generalized to taxonomic, phylogenetic, and functional diversities (Chiu & Chao, 2014; Chao, Chiu & Jost, 2014; Chao et al., 2019). Here, we use this framework to measure PD in a community.

### Phenological diversity assessment through the species-pairwise distance framework

The measure that we present here is based on a pairwise overlapping distance, following the same logic by Chao et al. (2014) in developing their functional diversity measure under the assumption that each species has specific phenological curves (Chao, Chiu & Jost, 2014; Chao et al., 2019). The distance we used is based on the Morisita-Horn index modified to measure the amount of overlap between pairs of phenological curves (Luna-Nieves et al., 2022). Let *O*_*ij*_ be the pairwise overlapping distance between the continuous phenological curves of the *i*-th and *j*-th species, defined as

**Figure 2:**
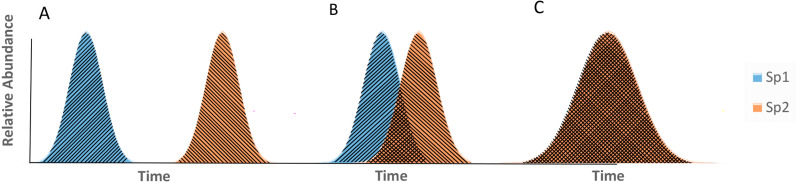
Theoretical overlap scenarios between pairs of phenological curves. (A) Non-overlapping phenological curves. (B) Phenological curves partially overlapping. (C) Fully overlapping phenological curves (the blue curve is behind the orange curve), species 1, species 2.

(1) \begin{eqnarray*}{o}_{ij}=1- \frac{2\int \nolimits {z}_{i}(t){z}_{j}(t)dt}{\int \nolimits {z}_{i}(t)^{2}dt+\int \nolimits {z}_{j}(t)^{2}dt} \end{eqnarray*}

where *z*_*i*_ and *z*_*j*_ are the smoothed continuous-over-time phenological curves of species *i* and *j*, respectively and the integral is calculated over the studied time interval. *O*_*ij*_ ranges in the [0, 1] interval, with *O*_*ij*_ = 1 when curves fully overlap (Fig. 2C), and *O*_*ij*_ = 0 when curves show no overlap (Fig. 2A). When completely overlapping in time (Fig. 2C), the species belong to the same phenological group; when they partially overlap, they partially belong to the same phenological group (Fig. 2B). A third case corresponds to the scenario in which the phenological curves do not overlap (Fig. 2A), which represents the existence of completely different phenological groups. Therefore, our approach to measuring phenological diversity is based on the pairwise overlap distance measured through the Morisita-Horn index (Horn, 1966; Garratt & Steinhorst, 1976).

Considering that both deterministic and stochastic variables contribute to the phenomena regulating phenology, similar phenological curves between pairs of species might suggest that they have similar environmental requirements, a common response to the same environmental cues, or similar evolutionary constraints. By contrast, dissimilar phenological curves suggest that the species have different environmental requirements or might simply reflect historical competitive displacement between species (Slagsvold, 1977; Baselga, Gómez-Rodríguez & Lobo, 2012). As the phenological curves may vary among time in position and shape. The overlapping area of the phenophase curves between pairs of species over the entire time series can provide an indirect and prospective measure of the interactions between phenophases only in the time frame measured. Because our framework of phenological diversity is based on the approach of “attribute diversity” (Chao, Chiu & Jost, 2014; Chao et al., 2019), which is a robust extension of Hill numbers, it can be applied to measure species traits and their diversity in orders of *q*. Within this framework, phenological Hill numbers (PD) can thus be interpreted as the pairwise phenological distance between species (in units of equally abundant and distinct species with distinct phenological group) occurring in an assemblage within the time interval over which it was measured.

To construct our phenological diversity measure we need to consider a standardizing factor: sum of the relative overlap of phenological curves, denoted as *Q.* This factor is calculated as follows (Chao, Chiu & Jost, 2014; Chao et al., 2019):

(2) \begin{eqnarray*}Q={\mathop{\sum \nolimits }\nolimits }_{i=1}^{S}{\mathop{\sum \nolimits }\nolimits }_{j=1}^{S}{O}_{ij}{p}_{i}{p}_{j},\end{eqnarray*}

where *O*_*ij*_ is calculated as in Eq. (1), and *p*_*i*_ is the relative intensity or abundance of the phenological event measured on species *i,* defined as

(3) \begin{eqnarray*}{p}_{i}= \frac{\int \nolimits {z}_{i} \left( t \right) dt}{\sum _{j=1}^{S}\int \nolimits {z}_{j} \left( t \right) dt} .\end{eqnarray*}

Finally, the phenological Hill numbers of order *q*, ^*q*^*PD*, is calculated as

(4) \begin{eqnarray*}\text{\hat qPD}=[{\mathop{\sum \nolimits }\nolimits }_{i=1}^{S}{\mathop{\sum \nolimits }\nolimits }_{j=1}^{S} \frac{{o}_{ij}}{Q} ({p}_{i}{p}_{j})^{q}]^{ \frac{1}{2\ast (1-q)} }.\end{eqnarray*}

Given that when *q* = 1 the exponent $ \frac{1}{2\ast (1-q)} $ is undefined, we decided to use the same approach as Chiu & Chao (2014). Therefore,

(5) \begin{eqnarray*}\text{\hat 1PD}=exp \left( - \frac{1}{2} {\mathop{\sum \nolimits }\nolimits }_{i=1}^{S}{\mathop{\sum \nolimits }\nolimits }_{j=1}^{S} \frac{{o}_{ij}}{Q} {p}_{i}{p}_{j}\log \nolimits ({p}_{i}{p}_{j}) \right) .\end{eqnarray*}

### Simulations and field data

**Figure 3:**
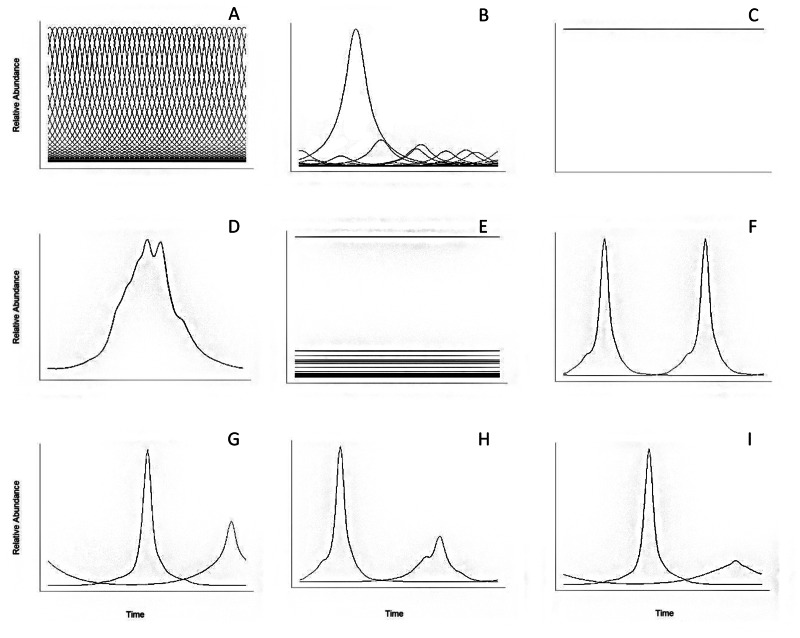
Graphs of the tested phenological diversity simulations. (A) *S* = 40, all species are equally distributed over time and the abundances are equal; (B) *S* = 40, all species are equally distributed over time and the abundances are unequal; (C) *S* = 40, all species are present all the time without abundance variation and abundances are equal; (D) *S* = 40, all species are present all the time with abundance variation and abundances are equal; (E) *S* = 40, all species are present all the time without abundance variation and abundances are unequal; (F) *S* = 2, species equally distributed over time with equal abundance; (G) *S* = 2, species not equally distributed over time with equal abundance; (H) *S* = 2, species equally distributed over time with unequal abundances; (I) *S* = 2, species not equally distributed over time with unequal abundances. A, B, C, D and E are modifications of the Madagascar amphibian community dataset.

To illustrate the utility of ^*q*^*PD*, we generated simulated data sets reflecting different community scenarios and applied our measure to these and one additional real data sets. Although these simulations are unlikely to occur in nature, they provide a glimpse into a real community phenological pattern (Fig. 3). Simulations vary in several community traits, such as: the degree of overlap, differential or even in intensity, and total number of species (*i.e., S*) (Fig. 3).

As previously explained, ^*q*^*PD* values represent the “phenological Hill numbers” and thus quantify the diversity of different phenological curves in a given assemblage. The contribution of the phenological curve of each species is considered unique and equally distinct from each other; thus, ^*q*^*PD* values always range from >0 (Fig. 3) to the total number of phenological curves measured (*S*, when there is no overlap at all between them). Therefore, phenological Hill numbers values have lower values when *q* increases.

**Figure 4:**
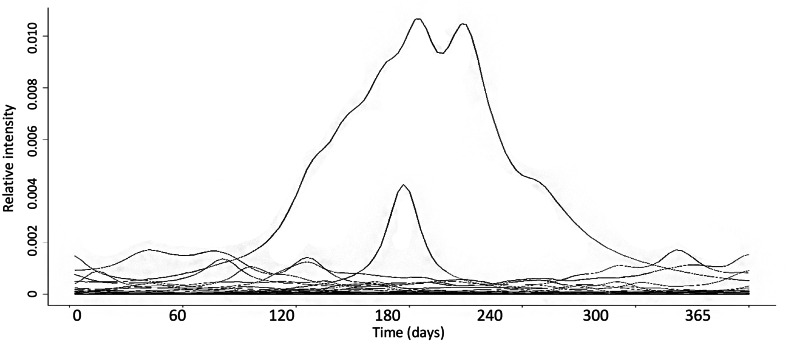
Wavelet transformed data of 120 sample points of an amphibian community in Madagascar over one year. Each line represents one phenological curve, *S* = 40.

In addition to the simulated data sets, we performed an analysis of ^*q*^*PD* based on field data for the breeding phenological activity of an amphibian community from Madagascar (Fig. 4; *S* = 40; time period = 360 days; frequency = daily; Heinermann et al., 2015).

All analyses were performed in R v. 4.1.2 (R Core Team, 2021) using the DescTools (Signorell et al., 2020) and wavScalogram (Benítez, Bolós & Ramírez, 2010) packages. We provide the script for calculating the phenological diversity measure in the Supplementary Material.

## Results

**Figure 5:**
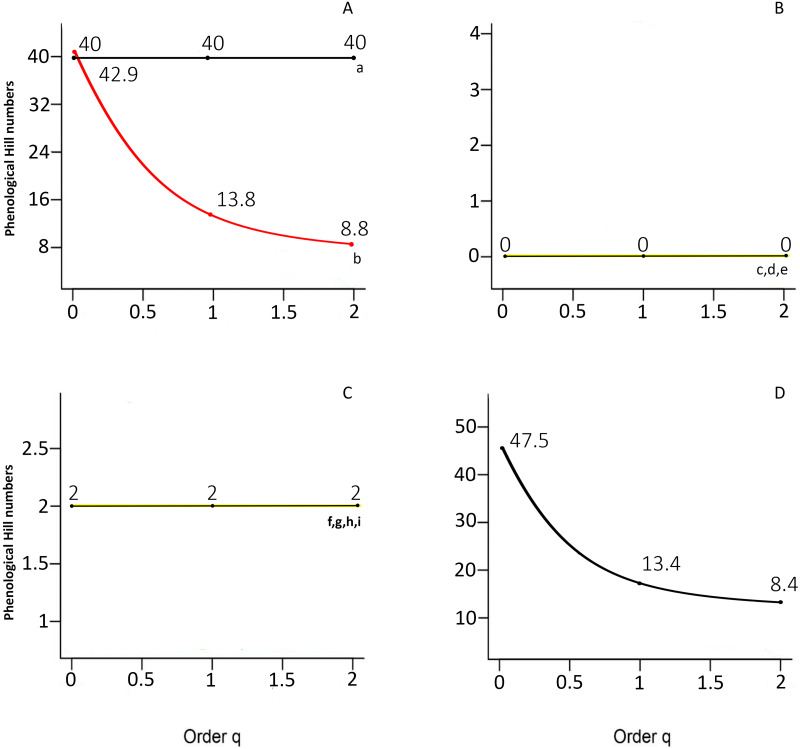
Phenological Hill numbers profiles as functions of *q* (0 ≤*q*≤ 2) for simulated (A-I) data and real data sets (amphibian community from Madagascar). (A) Case a, all species are equally distributed over time and have equal abundances; case b, all species are equally distributed over time and have unequal abundances. (B) Case c, all species are present all the time without abundance variation and equal abundances; case d, all species are present all the time with abundance variation among time but species present equal abundances; case e, all species are present all the time without abundance variation and unequal abundances. (C) Case f, species equally distributed over time with equal abundances; case g, species not equally distributed over time with equal abundances; case h, species equally distributed over time with unequal abundances; case i, species not equally distributed over time with unequal abundances. (D) Real data sets from Madagascar. Cases a, b, c, d, and e in (A) and (B) are modifications of the data from the amphibian community in Madagascar.

^*q*^*PD* values are illustrated as phenological Hill numbers profiles in Fig. 5. As expected, there is a tendency to decrease as *q* increases in cases with abundance variation (Fig. 5), and there is no decreasing pattern in simulations a, c, d, e, f, g, h and i. The absence of a decreasing pattern in these simulations corresponds to null variation in the intensity and overlapping area among species (Fig. 3).

The minimum values in simulations c and d reflect the lack of differentiation in the community phenological curves over time (Figs. 3C, 3D and 6B). The ^*q*^*PD* values were consistent with the homogeneous distribution of the intensity and concurrence of phenophases over time. The most diverse simulation was case a (Fig. 5A), where 40 species occur once in the year and the intensities of phenological curves are equal. By contrast, simulations c, d, e were the least diverse; with values of 0, where species occur all year-round with no differential phenophase intensities. Likewise, simulations f, g, h and i have low ^*q*^*PD* values because only two phenological curves were analyzed (Fig. 5C), and variation in the intensity and timing of the phenophases is apparent and the distance between two curves must be between 0 and 1 (Fig. 3). For simulations c, d and e (Fig. 5B), the distribution of the overlapping area among all phenological curves was the same. Overall, these results confirm that phenophase intensity is a phenological trait that directly affects ^*q*^PD values.

Figure 4 show the data for the phenological Hill numbers of the Madagascar amphibian community. ^0^*PD* for Madagascar was 47.5 (*S* = 40 species, 120-time sample points). There was a decreasing pattern as *q* increased, with ^2^*PD* values being reduced to 8.4.

## Discussion

Here, we propose a phenological diversity measure based on time series analysis and Hill numbers diversity theory that provides an efficient, readily interpretable and comparable measure that summarizes a key aspect of temporal biological diversity, namely the mean variety of phenological patterns observed in ecological communities. An initial step in developing our phenological diversity measure involves transforming data sets that are incomplete due to information gaps. Wavelet transform analysis proved useful for constructing a continuous phenological curve. Several studies have recommended the use of time series analysis in ecological and forestry studies (Senf et al., 2017; Li & Wu, 1995; Dale & Mah, 1998; Cho & Chon, 2006; Cazelles et al., 2008). This approach has been used for specific purposes such as population dynamics, disease transmission, and animal migration (Cho & Chon, 2006; Cazelles et al., 2008). Thus, incorporating time series in the study of phenology enhances our understanding of phenological diversity in communities, as it captures the continuous nature of phenology. A recent study using the Fourier transform showed that this tool can be used to detect periodical patterns in phenological cycles in long-term data (Bush et al., 2017), and this approach performed better than circular statistics (Morellato, Alberti & Hudson, 2010). However, these two approaches have not been used to model continuous phenological data. Likewise, we demonstrated that time series analysis can be used to model a continuous phenological curve from discrete or not fully continuous data; thus, there is a need to develop more robust theory aside from Fourier’s approach given that phenological patterns are not unimodal (*e.g.*, empirical orthogonal function, wavelets or Hilbert-Huang method; Cho & Chon, 2006; Cazelles et al., 2008; Bowman & Lees, 2013; Huang, 2014). Limitations of Fourier’s approach are primarily related to the information features of multi-scale functions at dominant intensities through the time series; in other words, Fourier analysis only provides information on the periods but not on their distribution over time. Consequently, Fourier transform is less applicable than wavelet analysis to nonlinear, nonstationary transient and scale-dependent phenomena such as natural processes characterized by high variability (Cees, 1999; Li & Wu, 1995). The specific advantage of wavelet analysis is that it considers the frequency of each time interval in the time series from small to large scales, which enables a more accurate calculation of nonlinear and nonstationary variables, such as phenological processes. As described in the Methods section, two parameters need to be specified: the scaling function and the attenuation threshold (*τ*). The scaling function relates to the nature of the time series data and the approach of the wavelet transform (Cazelles et al., 2008). Scaling functions are numerous and each one has a specific use. We used the Morlet function because of the lack of predictability in the biological data related to phenological traits (Percival & Walden, 2000) and its capacity for high-frequency resolution (Cazelles et al., 2008). The Daubechies scaling function is used in variables with fractal sequences or even in signal discontinuities (Akansu, Haddad & Çaglar, 1993). The Mexican hat scaling function is used in seismic signal patterns where variables show strong changes in the beginning and decrease over time (Zhou & Adeli, 2003). The latter features in temporal data are not observed in phenological information and therefore the use of these scaling functions is not warranted for this purpose.

The *τ* parameter reflects the rate of occurrence or disappearance of phenological traits over time. In nature, phenological processes generally occur gradually and continuously rather than abruptly and discretely; however, because of logistic restrictions, we are generally limited to collecting discrete records of phenological patterns. For example, the flowering of some cacti can occur in a single night (Petit, 2001), whereas the flowering of many tropical rainforest tree species tends to be gradual (Newstrom, Frankie & Baker, 1994; Brown & Hopkins, 1996). Regardless of the community, both examples take place in a continuous manner and are time scale-dependent. Therefore, the phenological curve slope can be adjusted depending on the nature of the phenological process, which directly affects the area of overlap of the pairwise distance and, consequently, how the phenological processes share time in the entire community. Although the standard value of us exploration of different approaches for defining *τ* to accurately describe the phenology of species is necessary given variability in the time and duration of phenophases among species. Until he standard value of 2 be used to permit comparisons to be made among different systems and studies. Changes in *τ* might modify ^*q*^*PD* values but not the diversity patterns in order of *q.* Future research on the standardization of values is needed to increase the comparability of results (see Materials and Methods for explanation; Percival & Walden, 2000).

The Morisita-Horn index was found to be appropriate for measuring the pairwise overlapping distance between phenological curves (Luna-Nieves et al., 2022). There are two other widely accepted measures (or metrics) of overlap between curves: the Jaccard overlap index (Smith, Solow & Preston, 1996; Yue & Clayton, 2005) and the Szymkiewicz-Simpson overlap coefficient (Ramos-Guajardo, González-Rodríguez & Colubi, 2020); however, these indices do not represent the intensity and the proportion of overlap of phenological curves. The Jaccard overlapping area index only accounts for the overlapping area of both samples but ignores the area outside of the overlapping area. The Szymkiewicz-Simpson index is based on the overlapping area and the area of the smaller phenological curve. Unlike these two indices, the Morisita-Horn Index is calculated by including both the overlapping and non-overlapping areas, thus making it a better tool for our purposes (Yue & Clayton, 2005). The temporal overlap of phenological curves over time reflects the temporal niche similarity between species and provides insight into the existence and magnitude of interactions such as competition, mutualism, and facilitation (Kochmer & Handel, 1986; Murdoch et al., 2002; Hodgson et al., 2011; Bergamo et al., 2018; Lane et al., 2018; Donohue, 2005).

The proposed measure of phenological diversity is an extended application of the principle of Hill numbers used to measure phylogenetic (Chao, Chiu & Jost, 2010) and functional diversity (Chiu & Chao, 2014; Chao, Chiu & Jost, 2014; Scheiner et al., 2017). The main advantage of our measure is its ability to provide a more objective and easy-to-interpret way for comparing the mean variety of phenological patterns observed across different studies (Chiu & Chao, 2014). Our approach retains the diversity patterns of order *q* as the rest of diversity measures encompassed by the Hill numbers framework does. In ecological terms, the phenological Hill numbers values can be interpreted as a quantification of the mean different ways in which the community displays phenological curves over time. When *q* = 0, the measure represents the mean number of phenological curves included in the analysis (richness) as long as they do not overlap. If they do, this number is reduced to 0 when they are all identical because the distance between phenological curves is zero. When *q* >0, the phenological curves shared by increasingly larger numbers of species are assigned higher weight in determining the phenological Hill numbers values. In practice, this means that phenological curves that are highly similar to each other in terms of time and intensity are grouped together, which ultimately translates into the mean effective number of phenological curves. Thus, regardless of the value of *q*, higher values of phenological Hill numbers represent a more heterogeneous arrangement and lower temporal overlap in the phenological curves within a community.

The simulations we performed represent the behavior of extreme scenarios of phenological Hill numbers. Results show that differences in intensity, overlapping area and variation in the number of phenological curves determine the values of phenological Hill numbers because this measure is directly linked to both variations in these variables and *q* values (Chao, Chiu & Jost, 2014). Specifically, the highest ^*q*^*PD* values correspond to data on phenophases evenly distributed over time, as has been shown in other diversity studies (*e.g.*, De Bello et al., 2009); this result is related to the degree of species evenness in the community and reflects the degree of concurrence in the phenological curves (Figs. 3A, 3B). Likewise, the lowest ^*q*^*PD* values correspond to the lowest degree of variation in the intensity of phenological curves and the constant presence of all phenological curves over time; from a biological perspective, there is no heterogeneity in this case, and all species have the same intensity in their phenological curves and occur in the same timeframe (Figs. 3C, 3D, 3E). When the intensity of phenological curves varies and the concurrence of curves remains constant (*e.g.*, Fig. 3A *vs.* Figs. 3B and 3C, or 3D *vs.* 3E), ^*q*^*PD* values decrease when *q* increases, demonstrating that the measure is sensitive to the intensity of the different phenological curves. We also demonstrate that ^*q*^*PD* values are closely related to the number of phenological curves measured (Fig. 3C). Therefore, our framework does provide a reliable measure of a key community attribute under different phenological scenarios. Phenological curves are constructed through signal processing by wavelet analysis and intensity or abundance data is needed; thus, our approach do not consider the presence/absence data frames and a modification of our framework must be developed due to the nature of binary data.

^*q*^*PD* can be successfully evaluated in In the case of the Madagascar amphibians data set, we calculated a maximum ^0^*PD* value of 47.5 for a group of 40 species monitored over time. As *q* increases, the effective number of the phenological curves became greatly reduced, implying that there are few ways in which phenological curves can occur when abundance is assigned more weight in estimating ^*q*^*PD*. In other words, there are between a mean of 13 (*q* = 1) or 8 (*q* = 2) distinct ways in which species partition their activity temporally throughout the studied year. This can be explained by the fact that amphibian activity is highly tied to rainfall patterns, and several species respond similarly to this factor (see Heinermann et al., 2015). Some amphibian species occur continuously throughout the year, whereas others only do so during a short period in the rainy season, throughout the entire rainy season, in the cold dry season, or under hot dry conditions. Overall, our analysis provided a robust measure that summarizes the diversity of these patterns. Nevertheless, ^0^*PD* values (47.5) are slightly larger than taxonomic diversity (40) because Q < 1. Thus, the distance measure used in this framework can alter diversity values but not the overall patterns.

The proposed method correctly incorporates the proportion of overlapping area and the intensity of phenological curves, making our phenological diversity measure consistent with the Hill numbers unified framework (Chao, Chiu & Jost, 2014; Chao et al., 2019). Moreover, the proposed framework enables any phenological phenomenon to be examined with any set of taxa at the community level. Nevertheless, two assumptions require consideration: (1) long time series data lead to a better modeling of continuous phenological curves (more than 25 is recommended) (Chamoli, Bansal & Dimri, 2007; Cazelles et al., 2008), and (2) our analysis assumes, due wavelet analysis, that phenology is a cyclical phenomenon and does not fit systems with non-cyclic patterns (Percival & Walden, 2000).

There is a need for more studies to examine phenological patterns, including long-term studies based on records of community phenological diversity patterns, to enhance our understanding of the environmental cues that underlie the structure of communities in different environments and how species share the temporal dimension in infra and supra annual scales, especially regarding the impacts of climate change and the problems associated with the increasing mismatch between phenophases of interacting species (Pau et al., 2011; Pérez-Ramos et al., 2020; Rafferty et al., 2013; Morente-López et al., 2018; Renner & Zohner, 2018). Specifically, our measure only summarizes the variability of a temporal trait of communities, and it should be tested and correlated with different environmental variables and phenophase measurements at different time frames and different taxonomic levels to further improve our understanding of the factors underlying the phenological patterns displayed by groups of species. Thus, our approach provides a new tool for measuring a single temporal attribute (PD) of communities and the correlations of this attribute with environmental variables can provide important insights that could aid conservation, restoration and management programs. Time series analysis should also be conducted under the assumption that the phenological data can be cyclical or not cyclical over time. Finally, we emphasize that ^*q*^*PD* is suitable for the analysis of massive datasets associated with the collection of phenological time series data and with any phenological process within a community.

## Conclusions

The phenological Hill numbers framework presented here produces simple and intuitive values for phenological diversity evaluation and thus can be widely applied to any taxon or community phenological traits using long-term data. Therefore, our measure has the properties of other diversity frameworks, and comparisons among studies using this same measure are possible. Phenological Hill numbers has important implications for the design of conservation and restoration programs that consider species and community patterns for the long-term persistence of biodiversity and *ad hoc* management.

##  Supplemental Information

10.7717/peerj.13412/supp-1 Supplemental Information 1 Annotated Code for Phenological Hill Numbers estimation

10.7717/peerj.13412/supp-2 Supplemental Information 2 Wavelet transformed data on Madagascar amphibian community

## Additional Information and Declarations

### Data Availability

The following information was supplied regarding data availability: The code for all simulations and analysis and the phenological curves are available in the Supplemental Files. The data from Madagascar amphibian community is available at DOI 10.1080/00222933.2015.1009513.
