# Supplementary material for: Quantifying phenological diversity: a framework based on Hill numbers theory
Source: PeerJ. 2022 May 12;10:e13412. doi: 10.7717/peerj.13412 (PMC9107786; doi:10.7717/peerj.13412)
Supplement: Supplemental Information 1 [file peerj-10-13412-s001.rtf]

library(DescTools)
library(wavScalogram)

signals <- read.csv("data")


dt <- 1
tau <- 2

n.time <- nrow(signals)
n.spp <- ncol(signals)
time <- 1:n.time
RelSpPhCumInt <- rep(NA, n.spp) # Relative Species Phenological Cumulative Intensity vector
wSpPhInt <- SpPhInt <- matrix(NA, nrow = n.time, ncol = n.spp) # Relative and absolute Species Phenological Intensity Continuous Patterns
for (i in 1:n.spp) {
  SpPhPat <- signals[, i]
  RelSpPhCumInt[i] <- sum(SpPhPat)
  if (sd(SpPhPat) == 0)
    SpPhInt[, i] <- SpPhPat/sum(SpPhPat)
  if (sd(SpPhPat) > 0) {
    wavelet <- cwt_wst(signal = SpPhPat, dt = dt, makefigure = FALSE) # wavelet analysis
    z <- abs(wavelet$coefs)^tau # wavelet-transformed signal
    SpPhInt[, i] <- rowSums(z)/sum(z) # modelled phenological continuous pattern
  }
}
RelSpPhCumInt <- RelSpPhCumInt/sum(RelSpPhCumInt)
for(i in 1:n.spp)
  wSpPhInt[, i] <- RelSpPhCumInt[i]*SpPhInt[, i]

 Plotting the community phenological patterns
for(i in 1:ncol(signals)) {
if (i == 1)
 plot (time, wSpPhInt[, i], type = "l", las = 1, bty = "l", xlab = "Time (yr)", ylab = "Intensity", ylim = c(0, max(wSpPhInt)))
if (i != 1)
lines(time, wSpPhInt[,i], col=viridis(40))
}

plot(time, wSpPhInt[, i], type = "l", ylab = "Intensity", xlab = "time")
for (i in 2:ncol(wSpPhInt))
  lines(time, wSpPhInt[, i], col = rainbow(ncol(wSpPhInt))[i])

# SECTION 2: Phenological Hill numbers diversity calculation

q <- seq(0, 2, length.out = 101)
qPhD <- rep(NA, ncol = length(q)) 

#q <- c(0, 1, 2) # these q-values can be changed if needed
#qPhD <- rep(NA, ncol = length(q)) 

Qij <- Oij <- matrix(NA, ncol = n.spp, nrow = n.spp)
for (i in 1:n.spp) {
  for (j in 1:n.spp) {
    Ii <- SpPhInt[, i]
    Ij <- SpPhInt[, j]
    Oij[i, j] <- 1-2*sum(Ii*Ij)/(sum(Ii^2) + sum(Ij^2)) # 1 - POI, POI as in Luna-Nieves et al. (2021)
    Qij[i, j] <- Oij[i, j]*RelSpPhCumInt[i]*RelSpPhCumInt[j]
  }
}
Q <- sum(Qij)
qPhDij <- array(NA, dim = c(length(q), n.spp, n.spp))
for (k in 1:length(q)) {
  if (Q == 0)
    qPhD[k] <- 0
  if (q[k] != 1 & Q != 0) {
    for (i in 1:n.spp)
      for (j in 1:n.spp)
        qPhDij[k, i, j] <- Oij[i, j]/Q*(RelSpPhCumInt[i]*RelSpPhCumInt[j])^q[k]
    qPhD[k] <- (sum(qPhDij[k, ,]))^(1/(2*(1-q[k]))) # Following eq.3 in Chiu & Chao (2014)
  }
  if (q[k] == 1 & Q != 0) {
    for (i in 1:n.spp) 
      for (j in 1:n.spp)
        qPhDij[k, i, j] <- Oij[i, j]*RelSpPhCumInt[i]*RelSpPhCumInt[j]/Q*log(RelSpPhCumInt[i]*RelSpPhCumInt[j])
    qPhD[k] <- exp(-0.5*sum(qPhDij[k, ,]))
  }
}

# References
# Chiu, C. H., & Chao, A. (2014). Distance-based functional diversity measures and their decomposition: a framework based on Hill numbers. PloS one, 9(7)
# Luna-Nieves, A. L., E. J. GonzÃ¡lez, J. CortÃ©s-Flores, G. Ibarra-ManrÃquez, A. Maldonado-Romo & J. A. Meave. In press. The interplay of exogenous and endogenous drivers of tropical dry forest tree phenology. Biotropica.
